# Diverse effects of a biosurfactant from *Rhodococcus ruber* IEGM 231 on the adhesion of resting and growing bacteria to polystyrene

**DOI:** 10.1186/s13568-016-0186-z

**Published:** 2016-02-18

**Authors:** Maria S. Kuyukina, Irena B. Ivshina, Irina O. Korshunova, Galina I. Stukova, Anastasiya V. Krivoruchko

**Affiliations:** Institute of Ecology and Genetics of Microorganisms, Russian Academy of Sciences, 13 Golev Street, Perm, 614081 Russia; Microbiology and Immunology Department, Perm State National Research University, 15 Bukirev Street, Perm, 614990 Russia

**Keywords:** Biosurfactant, *Rhodococcus ruber*, Bacterial adhesion, Cell hydrophobicity, Zeta-potential, Surface free energy, Atomic force microscopy

## Abstract

This study evaluated the effects of a trehalolipid biosurfactant produced by *Rhodococcus ruber* IEGM 231 on the bacterial adhesion and biofilm formation on the surface of polystyrene microplates. The adhesion of Gram-positive (*Arthrobacter simplex*, *Bacillus subtilis*, *Brevibacterium linens*, *Corynebacterium glutamicum*, *Micrococcus luteus*) and Gram-negative (*Escherichia coli*, *Pseudomonas fluorescencens*) bacteria correlated differently with the cell hydrophobicity and surface charge. In particular, exponentially growing bacterial cells with increased hydrophobicities adhered stronger to polystyrene compared to more hydrophilic stationary phase cells. Also, a moderate correlation (0.56) was found between zeta potential and adhesion values of actively growing bacteria, suggesting that less negatively charged cells adhered stronger to polystyrene. Efficient biosurfactant concentrations (10–100 mg/L) were determined, which selectively inhibited (up to 76 %) the adhesion of tested bacterial cultures, however without inhibiting their growth. The biosurfactant was more active against growing bacteria rather than resting cells, thus showing high biofilm-preventing properties. Contact angle measurements revealed more hydrophilic surface of the biosurfactant-covered polystyrene compared to bare polystyrene, which allowed less adhesion of hydrophobic bacteria. Furthermore, surface free-energy calculations showed a decrease in the Wan der Waals (γ^LW^) component and an increase in the acid-based (γ^AB^) component caused by the biosurfactant coating of polysterene. However, our results suggested that the biosurfactant inhibited the adhesion of bacteria independently on their surface charges. AFM scanning revealed three-type biosurfactant structures (micelles, cord-like assemblies and large vesicles) formed on glass, depending on concentrations used, that could lead to diverse anti-adhesive effects against different bacterial species.

## Introduction

Prevention and control of microbial biofilms are becoming of increasing importance in medical and industrial sectors since biofilms cause many recalcitrant patient infections in the clinical environment, spreading air- and foodborne pathogens and the fouling of industrial surfaces (Zezzi do Valle Gomes and Nitschke 2012; Bannat et al. 2014). Bacteria growing as a biofilm are more tolerant to antimicrobial treatments compared to planktonic cultures, thus requiring special eradication solutions (Davies 2003). An initial step of the biofilm formation is the adhesion of bacteria to surfaces, which depends on nutrient availability and environmental factors, as well as on the growth and physiological status of the bacterial population.

Biosurfactants, surface-active compounds produced by certain microorganisms were found to inhibit bacterial adhesion through the bioconditioning of surfaces or by interacting with bacterial cells and modifying their properties (Busscher et al. 1997; Meylheuc et al. 2001; Monteiro et al. 2011). Involvement of biosurfactants in microbial adhesion and desorption has been widely described (Neu 1996) and adsorption of biosurfactants to solid surfaces can be an effective strategy to reduce microbial adhesion and prevent the colonization by undesirable microorganisms (Bannat et al. 2014). Biosurfactants have also been reported to have antibacterial, antifungal and antiviral activities (Kitamoto et al. 2002; Cameotra and Makkar 2004; Díaz De Rienzo et al. 2015).

Non-toxic trehalolipid biosurfactants produced by non-pathogenic actinobacteria of the genus *Rhodococcus* have many industrially important properties summarized in several recent reviews (Kuyukina and Ivshina 2010; Franzetti et al. 2010; Kuyukina et al. 2015). We previously showed that a biosurfactant from *Rhodococcus ruber* IEGM 231 prevented the adhesion of human monocites to polystyrene, thus inhibiting their cytokine production, however without any cytotoxic effects (Gein et al. 2011). Also, this biosurfactant was shown to stimulate the adhesion of the producing strain to solid surfaces (Ivshina et al. 2013). Having such ambiguous results, we intended to study anti-adhesive and inhibitory effects of the biosurfactant against several strains of Gram-positive and Gram-negative bacteria.

It should be noted that anti-adhesive activity of biosurfactants is usually tested against non-growing (resting) bacteria (Busscher et al. 1997; Meylheuc et al. 2001, 2006; Rodrigues et al. 2006a, b; Das et al. 2009; Gudiña et al. 2010) and more rarely against growing biofilm-forming bacteria (Rivardo et al. 2009; Nithya et al. 2010; Monteiro et al. 2011). No comparative anti-adhesion experiments using both resting and growing bacterial cells were so far performed.

Bacterial cell surface properties, namely surface charge and hydrophobicity, play an important role in the initial adhesion steps (Loosdrecht et al. 1990; Vanhaecke et al. 1990; Walker et al. 2005). Numerous studies reported significant changes in physicochemical characteristics of bacterial cells during exponential and stationary growth phases that consequently alter their adhesive properties (Loosdrecht et al. 1987; Walker et al. 2005; Giaouris et al. 2009).

So, the aim of this study was to estimate the inhibitory effects of a crude biosurfactant produced by *R. ruber* IEGM 231 on growth and adhesion of actively growing and resting bacteria to polystyrene. The chemical structure and surface-active properties of this biosurfactant are described earlier (Kuyukina et al. 2001; Philp et al. 2002). The impact of surface conditioning by the biosurfactant on physicochemical characteristics of polystyrene, as well as the effect of the adsorbed biosurfactant layer on bacterial adhesion were studied in 96-microplates. In order to determine the influence of cell surface parameters on their adhesion and anti-adhesive activity of the biosurfactant, seven bacterial strains with different hydrophobicities and zeta potentials were selected, and their physicochemical properties were examined during both exponential and stationary growth phases. Atomic force images of bacteria surrounded by the biosurfactant adsorbed on glass were taken to reveal the biosurfactant assembly structures and their interactions with bacterial cells.

## Materials and methods

### Biosurfactant production

A *Rhodococcus ruber* IEGM 231 strain from the Regional Specialized Collection of Alkanotrophic Microorganisms, Perm, Russia (acronym IEGM, WFCC# 768, www.iegm.ru/iegmcol) was used for biosurfactant production. Bacteria were grown in *Rhodococcus* surfactant (RS) medium supplemented with 3 % (v/v) n-hexadecane at 160 rpm, 28 °C for 48 h (Ivshina et al. 2013). A crude biosurfactant was extracted from the bacterial culture with methyl tert-butyl ether (Kuyukina et al. 2001) and stored at −20 °C until use.

### Bacterial strains and growth conditions

For antibacterial and anti-adhesive assays, Gram-positive strains *Arthrobacter simplex* IEGM 667, *Bacillus subtilis* ATCC 6613, *Brevibacterium linens* IEGM 1830, *Corynebacterium glutamicum* IEGM 1861, and *Micrococcus luteus* IEGM 401, as well as Gram-negative strains *Escherichia coli* K-12 and *P. fluorescencens* NCIMB 9046 were used. Bacteria were grown in Luria–Bertani (LB) broth at 28 °C (or 37 °C for *E. coli*) and 160 rpm for 48 h (or 24 h for *E. coli*). Cells were harvested by centrifugation at 12,000 rpm for 3 min, washed twice with phosphate-buffered saline (PBS) and resuspended in PBS to the optical density at 630 nm (OD_630_) value of 1.5 (approximately 10^8^ cells/mL).

### Growth inhibition experiments

The antimicrobial activity of the crude biosurfactant against several bacterial strains was determined in 96-well flat-bottomed polystyrene microplates (Medpolymer, Russia). Briefly, tenfold serial dilutions of the biosurfactant in distilled water were made and ultrasonically treated (0.1A, 23 kHz) for 10 s to produce thin emulsions. 100 µL of sterile double strength medium (LB) were placed into the microplate wells and added with 50 µL of the prepared biosurfactant emulsions. Control wells were added with 50 µL of distilled water. Subsequently, 50 µL of bacterial suspensions were added into the wells. This resulted in the final biosurfactant concentrations 0.1, 1.0, 10, 100 or 1000 mg/L. Inoculated plates were covered and incubated in a Titramax 1000 vibrational platform incubator (Heidolph Instruments, Germany) at 150 rpm, 28 °C (or 37 °C for *E. coli*). After 48 h incubation, the OD_630_ was determined for each well with a Multiscan Ascent microplate photometer (Thermo Electron Corporation, Finland). Sixteen paralleled determinations were performed at all biosurfactant concentrations for each strain.

### Anti-adhesion experiments

The anti-adhesive activity of the crude biosurfactant against bacterial strains, which were used in growth inhibition experiments, was determined in 96-well flat-bottomed polystyrene microplates (Medpolymer, Russia) as described previously (Ivshina et al. 2013). Briefly, the biosurfactant was dissolved in isopropanol at concentrations 0.1, 1.0, 10, 100 or 1000 mg/L, and 200 μL of these solutions were added into microplate wells. The plates were then dried overnight under sterile air to evaporate isopropanol. In the anti-adhesion tests with resting cells, biosurfactant-coated and control (without biosurfactant) plates were filled up with 200 μL of bacterial suspensions and incubated stationary for 4 h at 28 °C (or 37 °C for *E. coli*). In the anti-adhesion tests with actively growing cells, biosurfactant-coated and control (without biosurfactant) plates were filled up with 180 μL of LB and 20 μL of bacterial suspensions. Inoculated plates were incubated in a Titramax 1000 incubator at 160 rpm for 48 h at 28 °C (or 37 °C for *E. coli*). After incubation, the OD_630_ was measured for each well with the Multiscan photometer, the liquid phase with non-adhered bacteria was carefully removed and the plates were washed twice with PBS using a Stat Fax^®^ 2600 washer (Awareness Technology Inc., USA). After washing, the remaining adhered bacteria were heat-fixed (60 °C, 1 h) and stained with 180 μL of 1 % (w/v) aqueous crystal violet solution. Followed 20-min staining at room temperature, the dye was removed and the plates were washed twice with PBS. Crystal violet incorporated into adhered cells was extracted with 180 μL of acetone:ethanol (70 %) mixture (1:4, v/v) for 5 min at 900 rpm, after that OD_630_ was measured with a Multiscan photometer. The percentages of adhered cells at different biosurfactant concentrations were calculated for each bacterial strain. Sixteen paralleled determinations were performed at all biosurfactant concentrations for each strain.

### Bacterial cell surface properties

The cell surface hydrophobicity of bacterial strains was assessed in the Bacterial Adhesion to Hydrocarbons (BATH) test (Rosenberg 1984). Bacterial strains pregrown in LB to the exponential (6 h for *E. coli* or 18 h for other bacterial strains) or stationary phase (24 h for *E. coli* or 56 h for other bacterial strains) were washed twice and suspended in PUM buffer (g/L: K_2_HPO_4_ × 3H_2_0, 2.2; KH_2_PO_4_, 7.26; urea, 1.8; MgSO_4_ × 7H_2_0, 0.2; pH 7.1) to the OD_600_ of 0.5. To 4.8 mL of the cell suspension in acid-washed test tube, 0.8 mL of *n*-hexadecane was added, and two phases were mixed by vortexing (Vortex FS 16 BioSan, Germany) for 2 min. After the mixture was allowed to separate for 1 h, the OD_600_ of the lower aqueous phase was measured using a Lambda EZ 201 spectrophotometer (Perkin Elmer, USA). The relative bacterial hydrophobicity was expressed as the percent loss in the OD_600_ relative to that of the initial cell suspension. All experiments were performed in six replicates.

The zeta potentials of bacterial cells were recorded by dynamic light scattering using a ZetaSizer Nano ZS analyzer (Malvern Instruments, UK) with Malvern Zetasizer v.2.2 software (Malvern Instruments, UK). Bacterial strains pregrown in LB to the exponential or stationary phase were washed and resuspended in 0.01 M KNO3 (pH 7.0) until OD_600_ of 0.15–0.2 was reached (Lambda EZ 201 spectrophotometer, Perkin Elmer, USA).

### Contact angle measurements of bare and biosurfactant-coated polystyrene

Contact angles with four different standard liquids (deionized water, glycerol, dimethylsulfoxide, and *n*-hexadecane) on bare and biosurfactant-covered polystyrene were determined using the sessile-drop technique. To obtain biosurfactant-covered polystyrene, the inner surface of the lid of 96-well polystyrene microplates (Medpolymer, Russia) was treated with a solution of 1000 mg/L of biosurfactant in isopropanol and drying for 15 min to evaporate the solvent under sterile air. A drop of tested liquid (5 µL for *n*-hexadecane or 50 µL for other liquids) was placed on the biosurfactant-treated or bare microplate lid and the contact angles were measured with an optical goniometer. For each liquid, at least 20 drops were measured on two independently biosurfactant-treated polystyrene surfaces. Contact angles of *n*-hexadecane, an apolar liquid, were used to calculate Lifshitz-van-der Waals free-energy values, whereas contact angles of water, glycerol, dimethylsulfoxide, having different polarities, were used to calculate electron-donating, electron-accepting and acid-based parameters (Boss et al. 1999).

### Atomic force microscopy (AFM) scanning

Bacterial cells were scanned using an Asylum MFP-3D-BIO atomic force microscope (Asylum Research, USA). Glass coverslips (50 × 24 × 0.16 mm) were covered with the biosurfactant by soaking with 100 or 1000 mg/L biosurfactant isopropanol solution and drying for 15 min to evaporate the solvent under sterile air. After drying, the coverslips were rinsed with deionized water and a drop (15–20 μl) of the bacterial cell suspension was placed on a coverslip (clean or covered with the biosurfactant), allowed to stand for 10 min and rinsed with deionized water to remove non-adhered cells. Alternatively, a bacterial suspension was added with the biosurfactant (100 or 1000 mg/L) sonicated emulsion and mixed vigorously, after which a drop of this mixture was placed on a coverslip, allowed to dry for 15 min, rinsed with deionized water and subjected to the AFM scanning in air using semi-contact (tapping) mode. Silicon cantilevers AC240TS (Olympus, Japan) with resonance frequencies of 50–90 kHz and spring constants of 0.5–4.4 N/m were used. Optimal scanning parameters determined experimentally to avoid cell damage or tip contamination were as following: scan rate, 0.4 line/s with 256 poins per line; set point, 650–720 mV; integral gain, 3. High resolution topographic (height) and amplitude images of the biosurfactant and bacterial cells were recorded and analyzed using the Igor Pro 6.22A (WaveMetrics, USA) software.

## Results

### Effect of *R. ruber* biosurfactant on bacterial growth

**Fig. 1:**
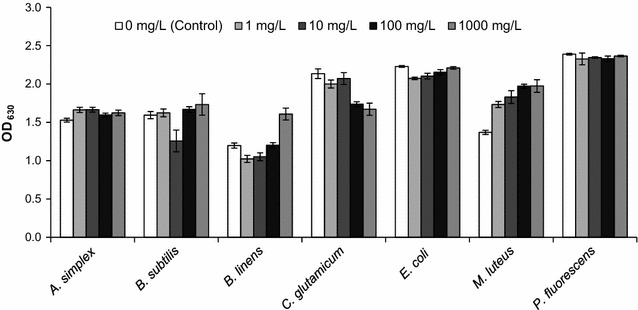
Effect of increasing biosurfactant concentrations on bacterial growth. Average OD_630_ value of 0.067 ± 0.008 was registered for the abiotic control (1000 mg/L biosurfactant without inoculation)

The crude biosurfactant showed the lack of antimicrobial activity against all bacterial strains tested (Fig. 1), except insignificant growth inhibition of *C.glutamicum* with highest tested concentrations (100 and 1000 mg/L). Moreover, some growth-promoting effects at the biosurfactant concentrations of 100 and 1000 mg/L were observed for *B.linens* and *M.luteus*.

### Adhesion of resting and growing bacteria to polystyrene

Tables 1 and 2 summarize the adhesion of bacterial strains to polystyrene with and without biosurfactant, depending on their physiological conditions (resting or growing cells). As seen from the first columns, resting cells of the most strains tested adhered weakly (5–13 %) to bare polystyrene (without biosurfactant), except *A. simplex* which showed relatively high adhesion activity (21 %). However, under growing conditions, the adhesive activities of *A. simplex*, *B. subtilis*, *E. coli* and *P. fluorescence* increased by 1.5–4 times, suggesting high biofilm-producing abilities of these strains, whereas the adhesion of *B. linens* and *M. luteus* decreased by 1.3–2 times, and the adhesion of *C. glutamicum* to polystyrene did not changed.

**Table 1:** Adhesion (%) of resting bacterial cells to polystyrene coated with biosurfactant at different concentrations

Bacterial strains	Biosurfactant concentration, mg/L					
	0	0.1	1	10	100	1000
*A. simplex* IEGM 667	21.4 ± 2.0	11.4 ± 1.1	10.9 ± 0.8	11.8 ± 1.3	11.4 ± 0.8	19.9 ± 2.9
*B. subtilis* ATCC 6613	7.7 ± 0.6	16.5 ± 2.2	12.0 ± 1.0	12.0 ± 1.3	14.6 ± 1.0	19.2 ± 2.1
*B. linens* IEGM 1830	12.6 ± 0.6	16.4 ± 0.9	16.5 ± 1.0	15.4 ± 1.1	14.7 ± 1.3	15.6 ± 1.6
*C. glutamicum* IEGM 1861	11.1 ± 0.5	11.4 ± 0.4	13.2 ± 0.7	13.8 ± 0.5	13.4 ± 0.4	9.6 ± 0.7
*E. coli* К-12	5.5 ± 0.3	5.8 ± 0.5	5.6 ± 0.4	5.5 ± 0.4	6.9 ± 0.5	11.7 ± 0.9
*M. luteus* IEGM 401	7.4 ± 0.6	6.9 ± 0.7	5.9 ± 0.2	6.7 ± 1.0	6.5 ± 0.4	11.1 ± 1.2
*P. fluorescens* NCIMB 9046	4.7 ± 0.3	4.4 ± 0.3	5.6 ± 0.5	4.1 ± 0.3	3.9 ± 0.2	5.2 ± 0.3

Here and in Table 2, means ± standard deviations of sixteen replicates are shown

**Table 2:** Adhesion (%) of growing bacterial cells to polystyrene coated with biosurfactant at different concentrations

Bacterial strains	Biosurfactant concentration, mg/L					
	0	0.1	1	10	100	1000
*A. simplex* IEGM 667	76.2 ± 4.9	96.9 ± 5.2	73.8 ± 4.1	81.8 ± 6.1	88.8 ± 5.5	57.2 ± 4.1
*B. subtilis* ATCC 6613	29.7 ± 3.2	31.9 ± 1.2	16.8 ± 3.1	7.1 ± 0.8	21.2 ± 2.6	53.1 ± 3.8
*B. linens* IEGM 1830	6.0 ± 0.3	10.5 ± 0.9	9.7 ± 0.7	7.0 ± 0.2	7.3 ± 0.3	5.3 ± 0.5
*C. glutamicum* IEGM 1861	11.9 ± 0.8	11.4 ± 0.6	9.3 ± 0.5	7.5 ± 0.4	8.1 ± 0.4	10.4 ± 0.8
*E. coli* К-12	8.3 ± 0.5	5.1 ± 0.3	4.8 ± 0.2	4.2 ± 0.2	4.7 ± 0.2	4.6 ± 0.1
*M. luteus* IEGM 401	5.5 ± 0.3	4.6 ± 0.2	4.3 ± 0.2	3.7 ± 0.2	3.9 ± 0.2	4.5 ± 0.3
*P. fluorescens* NCIMB 9046	8.8 ± 1.3	6.2 ± 0.3	6.8 ± 0.4	6.2 ± 0.4	6.9 ± 0.4	7.2 ± 0.6

### Biosurfactant affects differently the adhesion of resting and growing bacteria

**Fig. 2:**
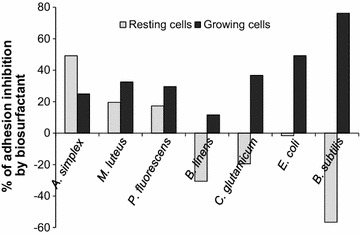
Diverse inhibiting and stimulating effects of the biosurfactant on resting and growing bacterial cell adhesion to polystyrene

A polystyrene coating with 0.1–100 mg/L biosurfactant affected negatively the attachment of resting *A. simplex* cells, leading to 50 % reduction of the adhesion. However, these biosurfactant concentrations did not prevent the adhesion of actively growing cells of the same strain, while 25 % reduction of adhered growing cells was observed at the maximal concentration (1 g/L) applied. For the most strains tested, there was no direct dose-dependent effect of the biosurfactant on the adhesion of resting cells. For instance, we observed 5–20 % reduction in the attachment of resting *M. luteus* and *P. fluorescence* cells to polystyrene covered with 0.1–100 mg/L biosurfactant, whereas their adhesion activities increased by 10–50 % at the maximal concentration of 1 g/L. Moreover, the adhesion of resting *B. subtilis, B. linens*, *C. glutamicum* and *E. coli* also increased with the increase in biosurfactant concentration. Thus, the biosurfactant at tested concentrations stimulated the adhesion of four bacterial strains and inhibited the adhesion of three other strains under non-growing conditions (Fig. 2).

Greater anti-adhesive effects of the biosurfactant were observed against actively growing *B. subtilis, C. glutamicum*, *E. coli*, *M. luteus* and *P. fluorescence* cells (Table 2), which allowed estimating the biosurfactant concentrations effectively inhibiting the biofilm formation by bacteria on polystyrene (Fig. 2). Indeed, the inhibition percentages for these 5 strains ranged from 30 to 76 % at the biosurfactant concentration of 10 mg/L. Applied at this concentration, the biosurfactant showed significant anti-adhesive potential against *B. subtilis* (76 %), *E. coli* (49 %) and *C. glutamicum* (36 %), while even at lower concentrations (0.1–1 mg/L) it was effective against *M. luteus* (15–20 %), *P. fluorescence* (11–30 %) and *E. coli* (34–42 %). However, 12–25 % inhibition of actively growing *A. simplex* and *B. linens* was observed only when the maximal biosurfactant concentration (1 g/L) was applied.

Summarizing, anti-adhesive effects of the *Rhodococcus* biosurfactant depended greatly on bacterial cultures tested and their physiological state. The biosurfactant inhibited more efficiently the adhesion of growing bacteria than resting cells.

### Influence of bacteria surface properties on their adhesion to polystyrene in the presence of biosurfactant

To understand better the mechanisms of interaction between bacterial cells and biosurfactant-coated polystyrene surfaces, we analyzed hydrophobic and electrokinetic properties of bacterial cells taken during exponential and stationary growth phases. Table 3 summarizes the results of cell surface hydrophobicity measured with the BATH test and bacterial surface charges (zeta potentials) measured as electrophoretic mobility. Based on BATH test results for non-growing (stationary phase) bacterial cells to *n*-hexadecane, three strains *A. simplex*, *B. linens* and *E. coli* were considered as strongly hydrophilic (<10 %), while two strains *M. luteus* and *P. fluorescence* were moderately hydrophilic (15–19 %), the *B. subtilis* strain was moderately hydrophobic (30 %) and *C. glutamicum* was strongly hydrophobic (>80 %) (Chae et al. 2006). However, exponentially growing cells of four strains from the total seven showed an increase in their hydrophobicity, particularly *A. simplex* (13.9 %), *B. subtilis* (51 %), *E. coli* (11.4 %) and *P. fluorescence* (24.1 %). High cell surface hydrophobicity of *C. glutamicum* (83–84 %) and relatively low hydrophobicities of *M. luteus* (19–21 %) and *B. linens* (10 %) were independent of the growth phase.

**Table 3:** Surface properties of bacterial strains depending on the growth phase

Bacterial strains	Surface hydrophobicity measured with the BATH test (%)		Zeta potential (mV)	
	Exponential	Stationary	Exponential	Stationary
*A. simplex* IEGM 667	13.9 ± 1.2	9.4 ± 1.3	−24.6 ± 2.1	−38.8 ± 2.3
*B. subtilis* ATCC 6613	51.0 ± 3.2	30.1 ± 2.8	−31.3 ± 2.0	−37.8 ± 2.4
*B. linens* IEGM 1830	10.2 ± 0.7	9.7 ± 0.8	−33.0 ± 3.2	−34.9 ± 2.2
*C. glutamicum* IEGM 1861	82.9 ± 6.2	84.2 ± 3.9	−35.4 ± 2.3	−33.1 ± 2.9
*E. coli* К-12	11.4 ± 0.8	6.6 ± 0.3	−47.9 ± 2.2	−45.1 ± 3.2
*M. luteus* IEGM 401	20.8 ± 1.0	18.6 ± 2.0	−36.9 ± 2.1	−33.3 ± 1.9
*P. fluorescens* NCIMB 9046	24.1 ± 2.1	14.8 ± 1.3	−26.4 ± 2.5	−20.5 ± 1.7

Zeta potential values of all strains were negative, ranging from −20.5 to −47.9 mV. Cells of *A. simplex* and *B. subtilis* in the exponential growth phase showed less negative surface charges compared to stationary phase cells. However, other strains demonstrated similar (*B. linens*, *C. glutamicum*) or slightly more negative (*E. coli*, *M. luteus*, *P. fluorescence*) zeta potentials of actively growing cells compared to stationary phase cells (see Table 3).

No significant correlation was found between hydrophobicity values and surface charges for resting or growing cells (corresponding correlation coefficients are 0.26 and −0.1) of the strains studied, however most hydrophilic *E. coli* had a highest negative charge (−45.1 mV) while strongly hydrophobic *C. glutamicum* had a lower negative charge (−33.1 mV).

Also, there was no linear correlation between the cell surface hydrophobicity and the adhesion of resting or growing bacteria to polystyrene (corresponding correlation coefficients are −0.01 and −0.11). However, a clear tendency was observed that exponentially growing cells of *A. simplex*, *B. subtilis*, *M. luteus* and *P. fluorescence* with increased hydrophobicities adhered stronger to polystyrene compared to more hydrophilic stationary phase cells of these strains. Whereas the strains whose hydrophobicities were independent on the growth phase demonstrated similar (*C. glutamicum*) or decreased (*B. linens*, *M. luteus*) adhesive activities during exponential growth.

The biosurfactant inhibited the adhesion of both hydrophobic and hydrophilic bacteria, while hydrophobic *C. glutamicum* and *B. subtilis* were inhibited only under growing conditions whereas these strains were stimulated under resting conditions. Hydrophilic *A. simplex*, *M. luteus* and *P. fluorescence* were inhibited in resting and growing states, while most hydrophilic *E. coli* was inhibited only in a growing state. Interestingly, a moderate correlation (0.41) was found between the hydrophobicity of exponentially growing bacteria and their adhesion inhibition by the biosurfactant. However, such correlation was not observed for the stationary phase (non-growing) bacteria.

Results of contact angle measurements (Table 4) indicated that the biosurfactant coating led to relative hydrophilization of the polystyrene surface (contact angles with deionized water and glycerol reduced from 88 and 79 to 86 and 75 degrees, correspondingly, whereas a contact angle with *n*-hexadecane increased from 10 to 41 degrees). Apparently, more hydrophilic surface would allow less bacterial adhesion. The surface free-energies calculated from the contact angles show a decrease in the Wan der Waals (γ^LW^) component and an increase in the acid-based (γ^AB^) component caused by the biosurfactant coating.

**Table 4:** Contact angles and surface free energy parameters of polystyrene with and without a biosurfactant adsorbed layer

Polystyrene surface	Contact angles (degree)				Parameters (mJ m^−2^)				
	Water	Glycerol	Hexadecane	Dimethyl-sulfoxide	γ	γ^LW^	γ^AB^	γ^−^	γ^+^
Uncovered	88 ± 2	79 ± 1	10 ± 2	51 ± 1	29.1	27.1	2.0	4.0	0.25
Covered with biosurfactant	86 ± 2	75 ± 2	41 ± 2	51 ± 1	27.2	21.2	5.0	4.3	1.45

There was no apparent influence of surface charges on the adhesion of resting bacteria to polystyrene (correlation coefficient is −0.29). However, for actively growing bacteria, a moderate correlation (0.56) was found between zeta potential and adhesion values. Also less negatively charged growing cells of *A. simplex* and *B. subtilis* adhered stronger to polystyrene. This was also true for biosurfactant-coated polystyrene surfaces (the correlation coefficient is 0.55). However, no correlation was found between the surface charges of bacterial cultures and their adhesion inhibition by the biosurfactant (corresponding correlation coefficients for resting and growing cells are 0.15 and −0.26). This finding suggested that the biosurfactant inhibited the adhesion of bacteria independently on their surface charges.

**Fig. 3:**
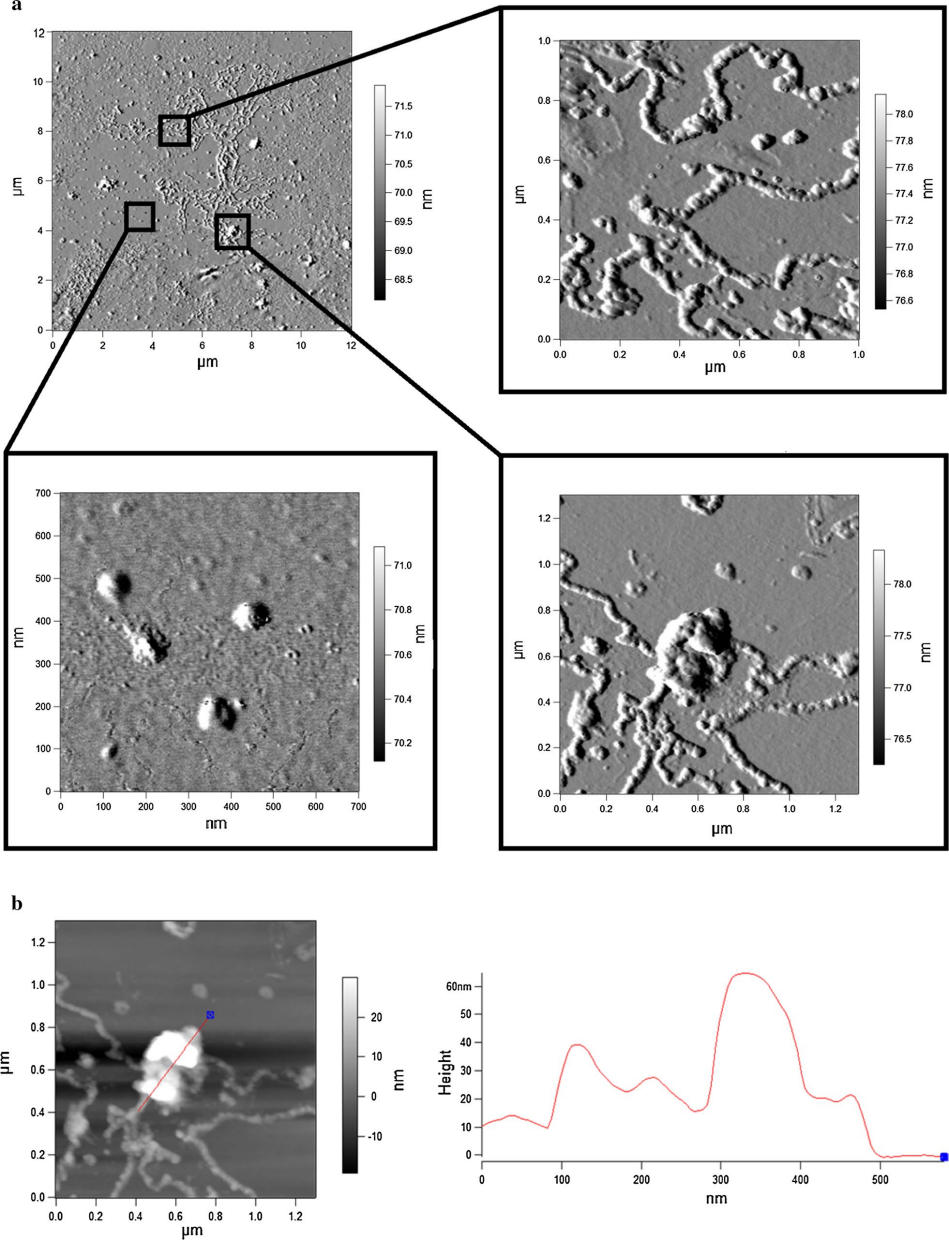
*AFM* (amplitude) images (**a**) and topographic (height) image with a profile (**b**) of the biosurfactant adsorbed on glass

AFM images of the biosurfactant adsorbed on glass revealed a formation of three type structures, depending of the biosurfactant concentration (Fig. 3a). In particular, micelle-like structures with an average diameter of 30–50 nm were observed at the low biosurfactant concentration (100 mg/L), which were either uniformly distributed on the glass surface or assembled in cord-like structures varying in length from 200 nm to 2 μm. While at higher concentration (1000 mg/L), the biosurfactant tended to aggregate into large irregular shaped vesicles ranging from 100 to 300 nm in diameters. A line scan drawn through the topographic image showed a height profile of cord-vesicle structures with corresponding heights of 10 and 40–60 nm (Fig. 3b). Such exaggerated two-dimensional sizes compared to heights were due to the tip pyramidal shape and strong biosurfactant adsorption to the glass surface resulted in the image convolution.

**Fig. 4:**
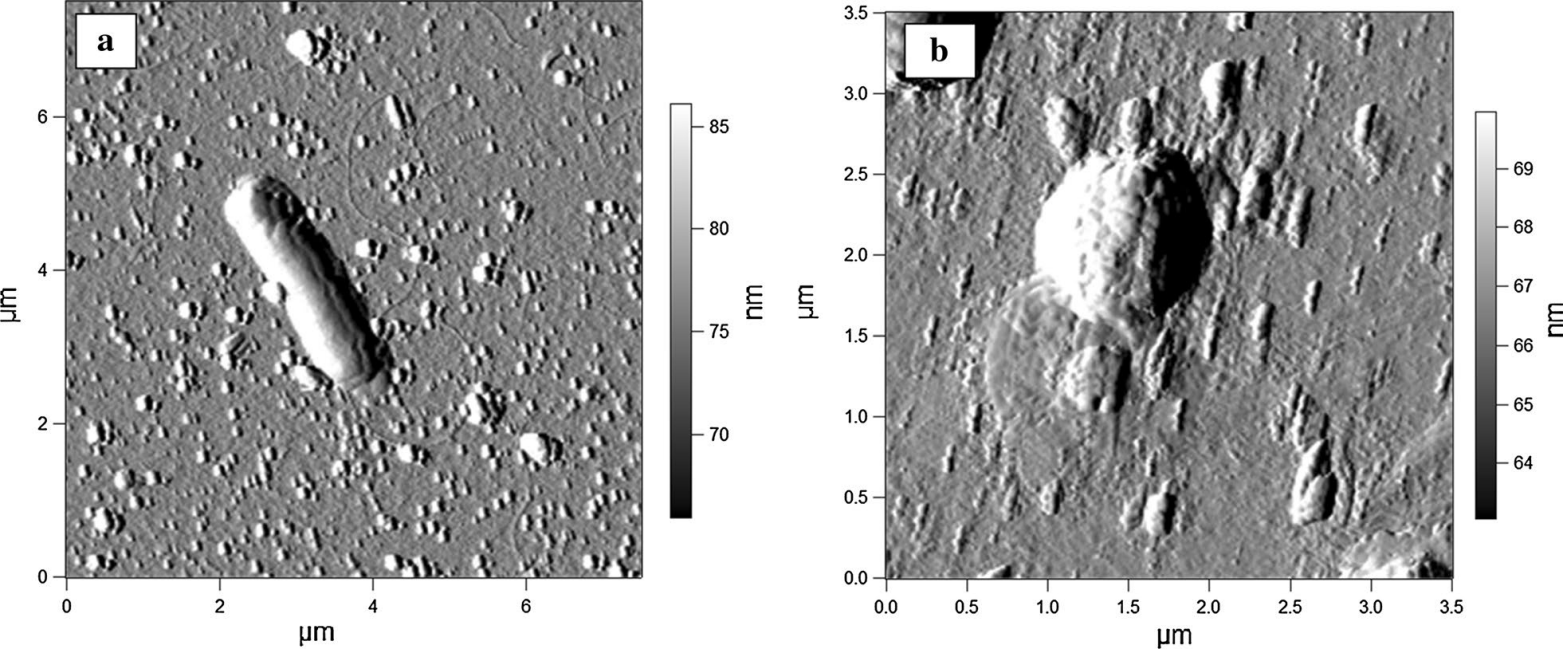
*AFM* (amplitude) images of *E. coli* (**a**) and *M. luteus* (**b**) cells on glass surrounded by biosurfactant micelles and vesicles

AFM images of bacterial cells surrounded by biosurfactant micelles and vesicles are shown in Fig. 4. It should be noted that corresponding images of these bacteria in the absence of biosurfactant revealed no differences (data not shown), thus indicating that both bacterial cell size and profile were not affected by the biosurfactant. This observation is consistent with the lack of antimicrobial activity of the biosurfactant revealed in growth experiments (see Fig. 1). Interestingly, we were not able to record cord-like biosurfactant structures on the glass surface in the presence of bacteria. It seems that bacterial cells disrupted cord-like assemblies (or prevented their formation), whereas more compact biosurfactant vesicles were resistant to this effect. Biosurfactant vesicles attached to *E. coli* and *M. luteus* cells can be seen in Fig. 4, suggesting that the *Rhodococcus* biosurfactant could interact with bacterial cells, thus influencing their interactions with the glass surface.

## Discussion

The biosurfactant from *R. ruber* IEGM 231 was found to affect differently the adhesion of resting and growing bacteria to polystyrene. Greater anti-adhesive effects of the biosurfactant were observed against actively growing *B. subtilis, C. glutamicum*, *E. coli*, *M. luteus* and *P. fluorescence* cells (Table 2), whereas resting cells of these strains were poorly inhibited or even stimulated by the biosurfactant. Such difference in the biosurfactant activity against resting and growing bacteria was not so far reported in the literature and it can be attributed to the changes in bacterial cell surface characteristics depending on the physiological state. It is known that an initial bacterial adhesion to solid substrates is greatly affected by two cellular factors, hydrophobicity and surface charge (Loosdrecht et al. 1990; Vanhaecke et al. 1990; Walker et al. 2005). So, we investigated these parameters of actively growing (exponential phase) and non-growing (stationary phase) bacteria (Table 3). As seen from Table 3, exponentially growing cells of four strains from the total seven showed an increase in their hydrophobicity compared to stationary phase cultures. Previous studies have also reported higher hydrophobicity of bacteria in the exponential phase (Loosdrecht et al. 1987; Heise and Gust 1999; Jana et al. 2000; Khemakhem et al. 2005; Gargiulo et al. 2007; Saini et al. 2011), while an opposite trend, i.e. hydrophobicity increase with the cell age was observed by other researchers (Allison et al. 1990; Nejidat et al. 2004; Walker et al. 2005; Zikmanis et al. 2007). In our experiments, exponentially growing cells of *A. simplex*, *B. subtilis*, *M. luteus* and *P. fluorescence* with increased hydrophobicities adhered stronger to polystyrene compared to more hydrophilic stationary phase cultures. These results suggested that hydrophobic interactions play an important role in the adhesion of these bacteria to hydrophobic polystyrene surface (Gallardo-Moreno et al. 2002). It should be noted that low adhesion of *B. Linens* and *M. Luteus* observed under growing conditions correlated with lower growth parameters of these strains compared to other cultures studied (see Table 1). Several authors indicated that bacterial adhesion to solid surfaces increases with the increase in the cell concentration up to some saturation limit probably due to kinetic (rate-limiting adsorption) or quorum sensing effects (Liu 1995; Sifri 2008).

Revealed higher anti-adhesive activity of the biosurfactant towards more hydrophobic actively growing bacterial cultures seems to be a result of changes in hydrophobic characteristics of the polystyrene surface (Table 4). Biosurfactant molecules due to their amphiphilic nature could bind to solid surfaces through polar or non-polar groups and produce a “conditioning film”, thus leading to the surface hydrophobization or hydrophilization (Neu 1996). Indeed, we previously used *Rhodococcus* biosurfactants for the hydrophobizing of initially hydrophilic sawdust to enhance the adhesion of the producing strain (Ivshina et al. 2013). In the present work, we applied the same biosurfactant for the opposite purpose—to reduce polystyrene hydrophobicity and prevent the adhesion of biofilm-forming bacteria.

As followed from the Table 3, surface charges of exponentially growing bacteria did not differ significantly from those of stationary phase cultures, except for *A. simplex* (zeta potential increased from **−**38.8 to **−**24.6 mV), *B. subtilis* (zeta potential increased from **−**37.8 to **−**31.3 mV) and *P. fluorescence* (zeta potential decreased from **−**20.5 to −26.4 mV). Since these exceptional strains adhered to polystyrene to a greater extent in the growing state, we have assumed that electrostatic interactions are also involved into the adhesion process (Giaouris et al. 2009). In particular, less negative zeta potentials of the *A. simplex* and *B. subtilis* exponential cultures (Table 3) could give a rise in the average percentage of cells adhering to the negatively charged polystyrene. Several authors suggested that for relatively hydrophilic organisms (like *A. Simplex* in our study) the main factor controlling the initial adhesion of bacteria is the surface charge (Loosdrecht et al. 1990; Giaouris et al. 2009). Our results provide some evidences for both electrostatic and hydrophobic forces involved into the bacterial adhesion to polystyrene but their contribution and interaction varied greatly depending on the particular culture.

Our results suggested that the biosurfactant inhibited the adhesion of bacteria independently on their surface charges, which is in agreement with a non-ionic nature of trehalolipid biosurfactants from *Rhodococcus* (Kuyukina and Ivshina 2010). However, higher inhibition of actively growing cells could partly be due to the changes in their zeta potentials to less negative values (Table 3). However, since growing cells of *A. simplex* were more negatively charged (−39 mV) compared to the resting cells (−25 mV), the biosurfactant was less active against growing cells of this bacterium.

It is worth noting that the revealed higher adhesion of actively growing bacteria to a solid surface compared to non-growing cultures should be considered, for example, when testing biofouling or corrosion-causing bacteria and new anti-corrosive coatings developed for nutritionally poor or rich environments (Tanji et al. 1999).

As seen from the Table 2, anti-adhesive action of the biosurfactant was not strongly dependent upon the concentration. A polystyrene coating with 10 mg/L biosurfactant effectively inhibited the adhesion of most cultures to polystyrene, resulting in 20–76 % reduction of attached bacterial cells. Moreover, even at lower concentrations (0.1–1 mg/L) it was effective against *M. luteus*, *P. fluorescence* and *E. coli*. However, inhibition of actively growing *A. simplex* and *B. linens* was observed only when the maximal biosurfactant concentration (1 g/L) was applied. Rivado et al. (2009) observed a strong concentration-dependent inhibition of the *E. coli* adhesion to polystyrene by a crude biosurfactant from *Bacillus licheniformis* V9T14, whereas other lipopeptide biosurfactant from *B. subtilis* V19T21 when used at low concentration (200 mg/L) showed maximal anti-adhesive activity against *Staphylococcus aureus*, which decreased at higher concentrations. Another lipopeptide biosurfactant from marine *Bacillus circulans* inhibited the bacterial adhesion to polysterene and disrupted biofilms formed by different bacteria in a concentration-dependent mode within 0.1–10 g/L (Das et al. 2009). Concentration-dependent anti-adhesive activities of the crude biosurfactant and glycolipid-rich fraction produced by *Streptococcus thermophilus* were revealed against several bacterial and yeast strains isolated from explanted voice prostheses (Rodrigues et al. 2006a). However, at high biosurfactant concentrations (20–40 g/L), a two-fold increase in concentration did not change the adhesion of *Streptococcus salivarius*, *Staphylococcus* spp. and *Rothia dentocariosa* to plastic. These findings could be explained by the saturation of polystyrene surface with biosurfactant molecules at threshold concentrations when further concentration increase does not result in the further changes of surface properties.

Obtained AFM images of the *R. Ruber* biosurfactant on glass coverslips revealed a formation of three type structures, depending of the biosurfactant concentration (Fig. 3). In particular, micelle-like structures with an average diameter of 30–50 nm were observed at the low biosurfactant concentration (100 mg/L), which were either uniformly distributed or assembled in long cord-like structures. While at higher concentration (1000 mg/L), the biosurfactant aggregated into large irregular shaped vesicles of 100–300 nm in diameters. Sánchez et al. (2007) have also observed three-size range rhamnolipid aggregates depending on the biosurfactant concentration, namely the 43–66 nm micelles coexisted with small (350–550 nm) aggregates at low concentrations, which were replaced by large (>1500 nm) aggregates at higher rhamnolipid concentrations. Many authors reported the spontaneous formation of higher order aggregates (having different shapes and sizes) from monodisperse micelles at increasing biosurfactant concentrations (Zhou et al. 2004; Pornsunthorntawee et al. 2009; Boettcher et al. 2010; Song et al. 2013) and the mechanisms of such micelle-to-vesicle transition are reviewed by Svenson (2004). Apparently, different *Rhodococcus* biosurfactant structures formed, depending on the concentration used, on the polystyrene surface would modify differently its physicochemical properties, thus leading to diverse anti-adhesive effects. Similarly, biosurfactants produced by *P. fluorescence* and *Lactobacillus helveticus* modified differently the stainless steel surface, resulting in diverse anti-adhesive effects against food-borne pathogenic bacteria (Meylheuc et al. 2006).

Another possible mechanism of the biosurfactant anti-adhesive action relates with its ability of binding to bacterial cells, altering their hydrophobic and electrochemical properties (Neu 1996; Monteiro et al. 2011). In this study, AFM images of *E. Coli* and *M. Luteus* on the biosurfactant-conditioned glass surface (Fig. 4) revealed the biosurfactant interactions with bacterial cells, which however did not affect the cell size and profile. This observation, coupled with the lack of growth-inhibiting activity of the biosurfactant (Fig. 1) suggested that the adhesion inhibition is mainly due to the interaction of biosurfactant molecules with bacterial cells or polystyrene surface, changing their surface properties, rather than its effect on the cell viability. Further research is required to clarify interactions of the biosurfactant with adherent bacterial cells, particularly concerning the formation of cord-like biosurfactant structures revealed by AFM on bare glass and absent in the presence of bacteria (Fig. 4).

In summary, anti-adhesive and biofilm-preventing effects of the *R. ruber* crude biosurfactant were revealed against Gram-positive and Gram-negative bacteria, which depended on strain properties (hydrophobicity and surface charge) and physiological stage. Efficient biosurfactant concentrations were found, which selectively inhibited the adhesion of tested bacterial cultures, however without inhibiting their growth. Interestingly, the biosurfactant was more active against growing bacteria rather than resting cells, thus showing high biofilm-preventing properties. Possible mechanisms of the biosurfactant anti-adhesive action were studied using hydrophobicity and zeta potential testing of bacterial cells, as well as contact angle measurements and AFM scanning of the biosurfactant.
